# Adult-onset KMT2B-related dystonia

**DOI:** 10.1093/braincomms/fcac276

**Published:** 2022-10-26

**Authors:** Edoardo Monfrini, Andrea Ciolfi, Francesco Cavallieri, Marco Ferilli, Paola Soliveri, Lucia Pedace, Roberto Erro, Francesca Del Sorbo, Franco Valzania, Valentina Fioravanti, Giovanni Cossu, Maria Pellegrini, Leonardo Salviati, Federica Invernizzi, Valentina Oppo, Daniela Murgia, Bruno Giometto, Marina Picillo, Barbara Garavaglia, Francesca Morgante, Marco Tartaglia, Miryam Carecchio, Alessio Di Fonzo

**Affiliations:** Dino Ferrari Center, Neuroscience Section, Department of Pathophysiology and Transplantation, University of Milan, Milan 20122, Italy; Foundation IRCCS Ca’ Granda Ospedale Maggiore Policlinico, Neurology Unit, Milan 20122, Italy; Genetics and Rare Diseases Research Division, Ospedale Pediatrico Bambino Gesù, IRCCS, Rome 00146, Italy; Neurology Unit, Neuromotor & Rehabilitation Department, Azienda USL-IRCCS di Reggio Emilia, Reggio Emilia 42124, Italy; Clinical and Experimental Medicine PhD Program, University of Modena and Reggio Emilia, Reggio Emilia 42124, Italy; Genetics and Rare Diseases Research Division, Ospedale Pediatrico Bambino Gesù, IRCCS, Rome 00146, Italy; Parkinson Institute, ASST G. Pini-CTO, Milan 20126, Italy; Fondazione Grigioni per il Morbo di Parkinson, Milan 20125, Italy; Department of Onco-Hematology, Cell Therapy, Gene Therapy and Hemopoietic Transplant, Ospedale Pediatrico Bambino Gesù, IRCCS, Rome 00165, Italy; Department of Medicine, Surgery and Dentistry ‘Scuola Medica Salernitana’, Neuroscience Section, University of Salerno, Baronissi, SA 84081, Italy; Parkinson Institute, ASST G. Pini-CTO, Milan 20126, Italy; Fondazione Grigioni per il Morbo di Parkinson, Milan 20125, Italy; Neurology Unit, Neuromotor & Rehabilitation Department, Azienda USL-IRCCS di Reggio Emilia, Reggio Emilia 42124, Italy; Neurology Unit, Neuromotor & Rehabilitation Department, Azienda USL-IRCCS di Reggio Emilia, Reggio Emilia 42124, Italy; Department of Neuroscience, Brotzu Hospital, Cagliari 09047, Italy; Neurology Unit, Trento Hospital, Azienda Provinciale per i Servizi Sanitari (APSS) di Trento, Trento 38122, Italy; Clinical Genetics Unit, Department of Woman and Child Health, University of Padova, Padova 35131, Italy; Medical Genetics and Neurogenetics Unit, Fondazione IRCCS Istituto Neurologico C. Besta, Milano 20126, Italy; Department of Neuroscience, Brotzu Hospital, Cagliari 09047, Italy; Department of Neuroscience, Brotzu Hospital, Cagliari 09047, Italy; Neurology Unit, Trento Hospital, Azienda Provinciale per i Servizi Sanitari (APSS) di Trento, Trento 38122, Italy; Department of Medicine, Surgery and Dentistry ‘Scuola Medica Salernitana’, Neuroscience Section, University of Salerno, Baronissi, SA 84081, Italy; Medical Genetics and Neurogenetics Unit, Fondazione IRCCS Istituto Neurologico C. Besta, Milano 20126, Italy; Neurosciences Research Centre, Molecular and Clinical Sciences Research Institute, St George's, University of London, London SW170RE, United Kingdom; Department of Experimental and Clinical Medicine, University of Messina, Messina 98122, Italy; Genetics and Rare Diseases Research Division, Ospedale Pediatrico Bambino Gesù, IRCCS, Rome 00146, Italy; Parkinson disease and Movement Disorders Unit, Department of Neuroscience, University of Padua, Padua 35131, Italy; Study Center for Neurodegeneration (CESNE), University of Padua, Padua 35131, Italy; Foundation IRCCS Ca’ Granda Ospedale Maggiore Policlinico, Neurology Unit, Milan 20122, Italy

**Keywords:** KMT2B, dystonia, hearing loss, DYT28, genetics

## Abstract

KMT2B-related dystonia (DYT-KMT2B, also known as DYT28) is an autosomal dominant neurological disorder characterized by varying combinations of generalized dystonia, psychomotor developmental delay, mild-to-moderate intellectual disability and short stature. Disease onset occurs typically before 10 years of age. We report the clinical and genetic findings of a series of subjects affected by adult-onset dystonia, hearing loss or intellectual disability carrying rare heterozygous *KMT2B* variants. Twelve cases from five unrelated families carrying four rare *KMT2B* missense variants predicted to impact protein function are described. Seven affected subjects presented with adult-onset focal or segmental dystonia, three developed isolated progressive hearing loss, and one displayed intellectual disability and short stature. Genome-wide DNA methylation profiling allowed to discriminate these adult-onset dystonia cases from controls and early-onset DYT-KMT2B patients. These findings document the relevance of *KMT2B* variants as a potential genetic determinant of adult-onset dystonia and prompt to further characterize *KMT2B* carriers investigating non-dystonic features.

## Teaser

Monfrini et al. report twelve cases from five unrelated families carrying four rare *KMT2B* missense variants. DNA methylation array analysis allowed the identification of a common pattern in these patients differentiating them from controls and early-onset KMT2B-related dystonic patients. This work widens the clinical spectrum associated with *KMT2B* missense variants, linking altered KMT2B function to adult-onset dystonia.

## Graphical Abstract

**Graphical abstract:**
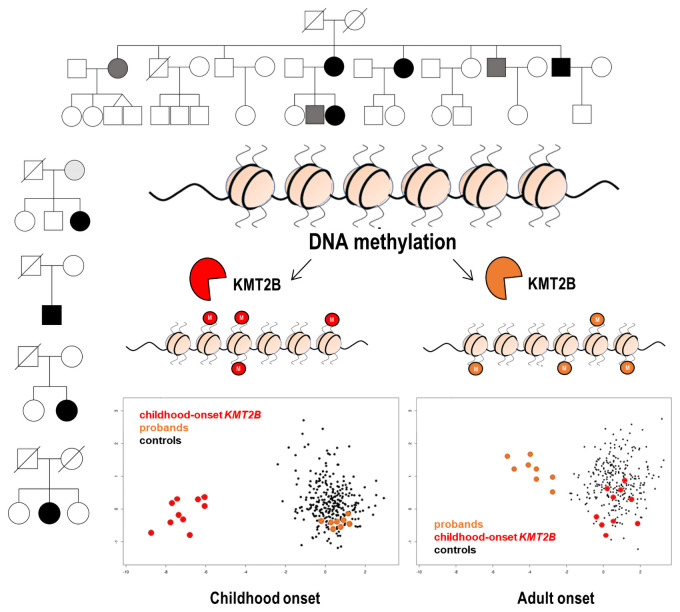

## Introduction

KMT2B-related dystonia (DYT-KMT2B, also known as dystonia 28, DYT28) is an autosomal dominant infantile-onset neurological disorder.^1,2^ Disease onset typically occurs before age 10; however, later onset has been seldom reported.^3,4^ DYT-KMT2B generally presents with a progressive course evolving from lower limb into generalized dystonia with prominent cranial, cervical, and bulbar involvement.^5,6^ Dystonia severity is variable and ranges from minor gait disturbances to wheelchair dependence.^5^ Psychomotor developmental delay, mild-to-moderate intellectual disability, and relative short stature can precede dystonia onset or be present in isolation as the only phenotypic signature in mutation carriers.^5^ Sensorineural hearing loss has also been rarely reported.^5^ Most of the DYT-KMT2B patients described to date harbour a *de novo KMT2B* variant, that can show incomplete penetrance within the same family.^5^

**Figure 1:**
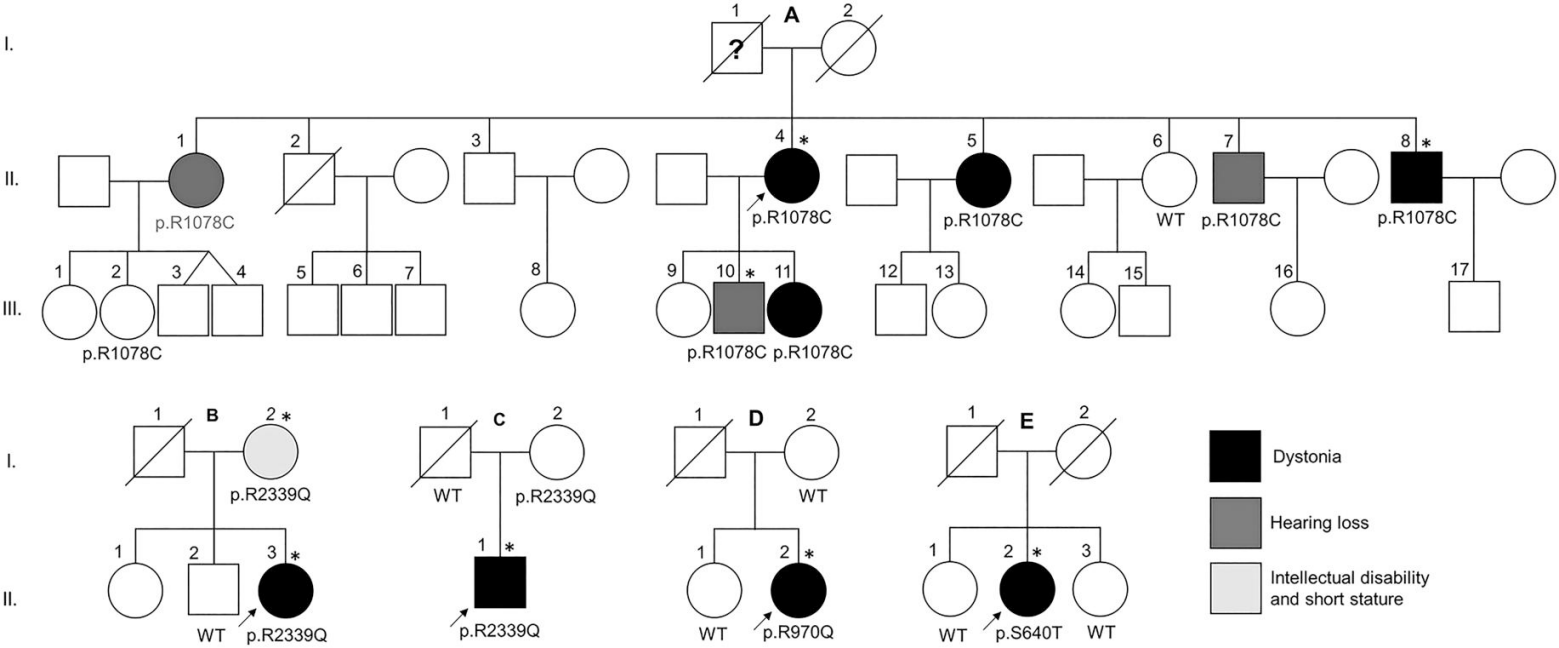
**Family pedigrees and *KMT2B* variants segregation**. Families of the probands A.II.4, B.II.3, C.II.1, D.II.2 and E.II.1. Black, dark grey and light grey fillings indicate affected status by dystonia, hearing loss and short stature/intellectual disability, respectively. Asterisks indicate the subjects that underwent DNAm array analysis.

In this clinical and genetic report, we present twelve cases from five unrelated families (Fig. 1) carrying four rare functionally relevant *KMT2B* variants who presented with adult-onset dystonia or non-dystonic phenotypes including progressive hearing loss, intellectual disability and short stature.

## Materials and methods

### Genetic analysis

Genomic DNA was extracted from peripheral venous blood by standard salting-out procedures. Probands’ DNA was analysed by WES using the Nextera Rapid Capture Exome Library kit (Illumina, San Diego, CA, USA) and the Illumina NextSeq500 platform (Illumina), according to the manufacturer instructions. Reads were aligned against the human reference genome (hg38) using BWA and variant calling was performed with GATK4. A virtual gene panel targeted for genetic dystonias (Supplementary material) was used to filter WES data. The candidate *KMT2B* variants were validated by Sanger sequencing and tested in available relatives.

### DNA methylation analysis

Genome-wide DNA methylation (DNAm) analysis was performed on peripheral blood DNA using Infinium Methylation EPIC BeadChip array (Illumina), according to the manufacturer’s protocol. Data pre-processing and normalization starting from IDAT files were performed as previously described.^7–9^ The DNAm profiles of eight affected cases (A.II.4, A.III.10, A.II.8, B.II.3, B.I.2, C.II.1, D.II.2, E.II.2) were compared with those from an in-house database including ∼300 samples from healthy individuals and patients with different rare neurodevelopmental disorders, using the established DNAm signature for early-onset DYT-KMT2B by means of multidimensional scaling (MDS), considering the pair-wise Euclidean distances between samples. The training of the SVM classifier was performed with a linear kernel using the e1071 R package (V.1.7) using nu-classification option. To determine the best hyperparameter and to measure the accuracy of the model, the whole data set was split in a training set (75% of samples) and a test set (25% of samples), and a 5-fold cross-validation was performed during the training process. Scores from SVM classifier below 0.25 were considered as control samples, from 0.25 to 0.5 were considered inconclusive findings, whereas > 0.5 indicated predicted pathogenic variants. The selected cases were then analyzed together with 27 age- and sex-matched controls to identify the most informative differentially methylated probes.^7–9^ The selected subset of differentially methylated probes (Supplementary Table 2) was validated using the entire in-house data set by MDS analysis.

### Data availability

The Ethics Committee of the IRCCS Foundation Ca’ Granda Ospedale Maggiore Policlinico (Milan, Italy) approved the study. Written informed consent for publication of clinical details, clinical images, and video recording were obtained from all involved subjects. Due to privacy and ethical concerns, supporting genetic and epigenetic data can only be made available to bona fide researchers subject to a non-disclosure agreement.

## Results

### Clinical features

#### Family A

Family A originated from a Sardinian village. No consanguinity was reported. All family members had normal birth and psychomotor development. At age 69, A.II.4 developed severe eyelid apraxia and pretarsal blepharospasm that led to functional blindness (Video 1). Local injections of botulinum toxin produced some benefit, but the duration of relief was less than two months. Subject A.II.5 developed at the age of 62 a dystonic head tremor and action tremor in both hands, which remained stable over the years. At age 56, Subject A.II.8 developed severe blepharospasm; two years later, he developed cervical dystonia (right torticollis and laterocollis) and neck pain (Video 1). Local injections of botulinum toxin were effective. Brain MRI was unremarkable in all three siblings. Subjects A.II.1 and A.II.7 developed isolated hearing loss in infancy. Their father (A.I.1) was reported to have developed a tremor, diagnosed as Parkinson’s disease, at the age of 50, and died at age 93. Two children of the affected Subject A.II.4 were tested (A.III.10 and A.III.11) since A.III.10 developed hearing loss in childhood, and A.III.11 displayed abnormal hand posture when writing since childhood (writer’s cramp).

#### Family B

Subject *B.II.3* is a 36-year-old female born prematurely at Week 36 of gestation. Psychomotor development and school performance were normal. At the age of 19 years, she complained of a subacute motor impairment affecting the left upper limb with the development of a fixed dystonic posture within a few months. In the subsequent year, dystonia spread to the left lower limb and speech became progressively rhinolalic. The motor phenotype thereafter stabilized, whereas a progressive disturbance of ocular movements developed. By age 23, horizontal saccades were slow and reduced in amplitude, and in two years she developed progressive oculomotor apraxia on the horizontal gaze (Video 1). Brain MRI at age of 23 was normal. No family history of dystonia was reported; however, her mother (subject B.I.2) presented mild intellectual disability (Total IQ 58—WEIS-IV) and short stature (126 cm).

#### Family C

Subject C.II.1 was born at term after a difficult delivery. Since childhood, he presented writing difficulties and his handwriting was poorly understandable. At the age of 34, he developed dystonic posture and tremor of the upper right limb. Neurological examination at the age of 49 showed dystonic movements and tremor of the right upper limb. These were induced by action and modulated by position. Brain MRI showed mild enlargement of the left lateral ventricle. I-123-Ioflupane SPECT was normal. Trihexyphenidyl, levodopa and tetrabenazine were not beneficial. He achieved some improvement with botulinum toxin.

#### Subject D.II.2

Subject D.II.2 was born at term and had normal psychomotor development. No family history of neurological diseases was present. At the age of 23, the patient progressively developed spasmodic dysphonia with dysarthric speech, a strained and strangled voice, and voice breaks during speaking. From the age of 29, a bilateral hand dystonic tremor developed, prevalent on the right side, especially during writing; dystonia then spread to the oromandibular region and cervical region; laryngeal impairment worsened leading to a significant decrease in speech intelligibility (Video 1). Anticholinergics did not bring any benefit. Botulinum toxin injections led to moderate improvement of dystonia. Brain MRI performed at the age of 44 showed mild brain atrophy and mild symmetrical pallidal hypointensity on T_2_*-weighted sequences.

#### Subject E.II.2

Subject E.II.2 was born at term after an uneventful pregnancy. No familial history of neurological disorders was present. At the age of 43, she progressively developed upper right arm focal dystonia. Cervical spine and brain MRI were unremarkable. Several drugs (anticholinergics, benzodiazepines, baclofen, tetrabenazine, and botulinum toxin injections) were ineffective. Neurological examination at age of 59 disclosed dystonic posture of the right upper limb with abduction of the arm and flexion–pronation of the forearm that worsened on action. The remainder neurological examination was normal.

### Genetic analysis

A virtual gene panel for dystonia was applied on WES data of probands A.II.4, B.II.3, C.II.1, D.II.2 and E.II.2. Four rare heterozygous *KMT2B* variants were identified: c.3232C > T in subject A.II.4, c.7016G > A in subjects B.II.3 and C.II.1, c.2909G > A in subject D.II.2 and c.1918T > A in subject E.II.2. No pathogenic or rare variants were found in other dystonia genes. *KMT2B* variants validation and segregation analyses in the families of the probands were performed by Sanger sequencing (Table 1).

**Table 1:** Clinical and genetic features of KMT2B variants carriers

Family	Subject	Dystonia	Age at onset (dystonia)	Dystonia onset localization	Other localizations	Dystonic tremor	Short stature	Hearing loss	Intellectual disability	*KMT2B* variant
A	II.1	−	NA	NA	NA	−	−	+	−	c.3232C > T (p.R1078C)
A	II.4	+	69	Blepharospasm	−	−	−	−	−	c.3232C > T (p.R1078C)
A	II.5	+	62	Head	Both arms	+	−	−	−	c.3232C > T (p.R1078C)
A	II.7	−	NA	NA	NA	−	−	+	−	c.3232C > T (p.R1078C)
A	II.8	+	56	Blepharospasm	Neck	−	−	−	−	c.3232C > T (p.R1078C)
A	III.10	−	NA	NA	NA	−	−	+	−	c.3232C > T (p.R1078C)
A	III.11	+	6	Hand	−	−	−	−	−	c.3232C > T (p.R1078C)
B	I.2	−	NA	NA	NA	−	+	−	+	c.7016G > A (p.R2339Q)
B	II.3	+	19	Left arm	Larynx, left lower limb	−	−	−	−	c.7016G > A (p.R2339Q**)**
C	II.1	+	34	Both arms	−	+	−	−	−	c.7016G > A (p.R2339Q)
D	II.2	+	23	Larynx	Face, both arms	+	−	−	−	c.2909G > A (p.R970Q)
E	II.2	+	43	Right arm	−	−	−	−	−	c.1918T > A (p.S640T)

The c.3232C > T (p.R1078C) is absent from population databases, but it has been already reported by Ciolfi A. et al.^8^ It is predicted pathogenic by all *in silico* prediction tools. Arginine1078 is conserved among orthologues. Previously performed functional analyses indicated that the genome-wide peripheral blood DNAm profile associated with this variant was different from that of *KMT2B* mutations causing childhood-onset DYT-KMT2B, suggesting a different functional impact of this missense change.^8^ The same variant was found in all the affected members of the family (A.II.5, A.II.7, A.II.8, A.III.10 and A.III.11), indicating co-segregation of the c.3232C > T with the disease (dystonia or hearing loss). The affected subject A.II.1 was an obligate carrier of the variant. One asymptomatic 50-years-old female carrier was also found (A.III.2).

The c.7016G > A (p.R2339Q) is a very rare known variant (dbSNP: rs751409145). Its allele frequency in gnomAD is 0.0001442. This variant is predicted benign by most in silico prediction tools. Genetic analysis of family B showed that this variant was carried also by the affected parent (i.e. B.I.2). Therefore, segregation analysis supported its pathogenic role in family B. Subject C.I.2 was carrier of the variant and reportedly asymptomatic.

The c.2909G > A (p.R970Q) is a known very rare variant (dbSNP: rs780053167) with an allele frequency of 0.000004024 in gnomAD. The amino acid Arginine970 is highly conserved among orthologues. Most in silico tools predict this variant to be pathogenic. The unaffected mother and the sister of the proband did not carry this variant. The DNA of the neurologically unaffected father was not available as he was already deceased from other causes.

The c.1918T > A (p.S640T) is a very rare missense variant (dbSNP: rs771667749) never reported in dystonic patients so far. The gnomAD allele frequency is 0.00005. This variant is predicted benign by most in silico prediction tools. Segregation analysis demonstrated that the healthy sisters of the proband did not carry this variant.

### Genome-wide DNAm array analyses

**Figure 2:**
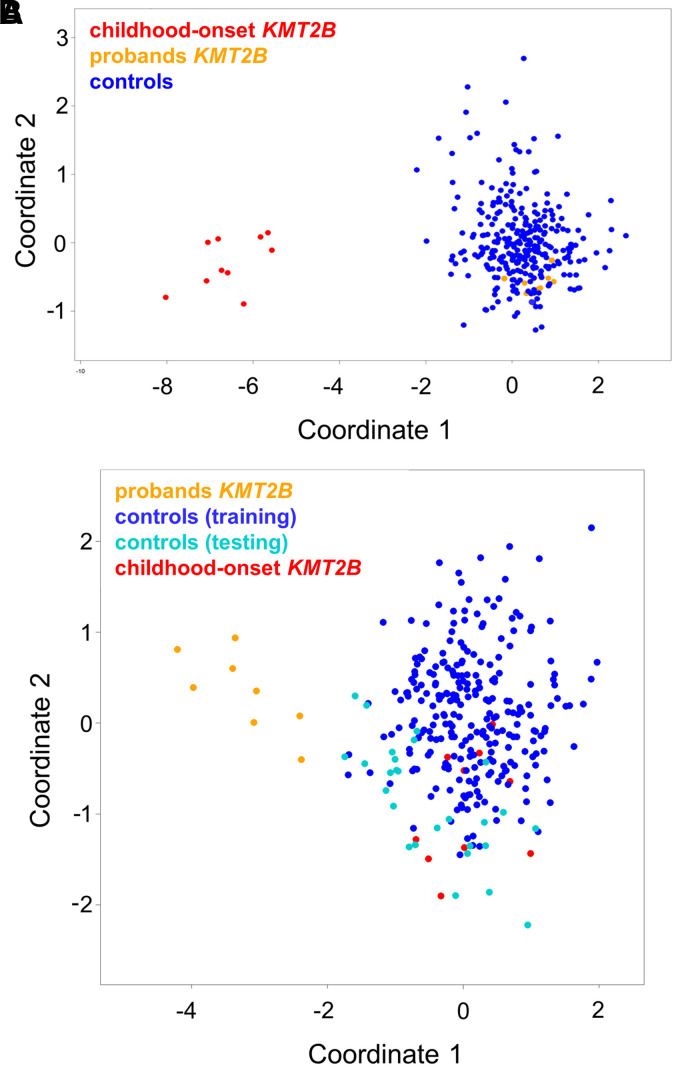
**DNAm array analyses**. (**A**) DNAm profiles in patients with *KMT2B* variants associated with late-onset dystonia differ from those characterizing childhood-onset DYT-KMT2B (DYT28). MDS plot is used to classify the presently identified *KMT2B* missense variants (orange) with respect to *KMT2B* variants causing childhood-onset DYT-KMT2B (red) and an in-house DNAm data set including ∼300 healthy individuals and subjects with rare neurodevelopmental disorders (blue). (**B**) Genome-wide DNAm analysis was able to cluster cases with late-onset dystonia (*n* = 6), hearing loss (*n* = 1), and intellectual disability-short stature (*n* = 1) carrying heterozygous *KMT2B* missense variants (orange) from childhood-onset DYT-KMT2B (red) and control samples (blue), by MDS analysis. The age-and sex-matched controls selected to identify differentially methylated probes are highlighted in black.

To investigate the functional relevance of the identified *KMT2B* variants associated with late-onset dystonia, hearing loss, and short stature-intellectual disability, a genome-wide DNAm analysis by means of EPIC array was performed as previously described.^8^ As a first step, we analyzed the DNAm profiles of eight affected individuals (A.II.4, A.III.10, A.II.8, B.II.3, B.I.2, C.II.1, D.II.2, E.II.2), six affected by adult-onset dystonia, one by hearing loss, and one by intellectual disability and short stature, carrying the four identified missense changes in the context of the episignature characterizing early-onset DYT-KMT2B by MDS analysis (Fig. 2A).^8^ The clustering of the eight tested cases, localizing far from the early-onset DYT-KMT2B group and within the controls, did not support the functional equivalence of the presently identified *KMT2B* missense variants with those causing early-onset DYT-KMT2B. This finding was confirmed by the low scores obtained by the SVM classifier (Supplementary Table 1). To explore the occurrence of a distinctive DNAm pattern shared by the patients reported here, these cases were compared with 27 age- and sex-matched unaffected subjects by linear modelling,^8,9^ allowing to identify 175 independent differentially methylated probes (Supplementary Table 2). This probe-set was validated considering DNAm data referred to 270 unaffected controls and patients with rare neurodevelopmental disorders by MDS analysis, which confirmed the occurrence of a separate cluster including all subjects with late-onset dystonia, hearing loss, and short stature-intellectual disability (Fig. 2). These specific probes did not overlap with early-onset DYT-KMT2B episignature, suggesting a different molecular mechanism underlying this late-onset phenotype.

## Discussion

In this report, we present 12 subjects from five unrelated families carrying four rare functionally relevant *KMT2B* missense variants, identified by a virtual dystonia gene panel derived from WES data.

The possible pathogenic role of the identified *KMT2B* variants is supported by the association with a consistent disease phenotype and their very low frequency in genetic databases. Moreover, the p.R1078C and p.R2339Q co-segregated with the phenotypes in families A and B, respectively. In particular, p.R1078C segregation in a large adult-onset dystonia family strongly supports its claim of pathogenicity. *In silico* tools predicted a likely pathogenic effect for only two of these variants (i.e. p.R970Q and p.R1078C). One of these variants has been already reported (i.e. p.R1078C) and its genome-wide DNAm episignature profiling indicated a different impact compared with the highly deleterious *KMT2B* variants causing childhood-onset DYT-KMT2B. The present DNAm analyses confirm the differential behaviour of the identified missense *KMT2B* variants associated with late-onset dystonia, although they provide evidence of a distinctive DNAm pattern, which suggests their functional and clinical relevance.

These clinical, genetic and functional findings support the hypothesis that *KMT2B* missense variants with mild pathogenic effect on methylation profile, and not predicted deleterious in silico, could be associated with later disease onset and lower penetrance, considering that two asymptomatic carriers were also found. In line with this reasoning, previous literature supported the hypothesis of an inverse correlation between mutation severity and age at onset.^10^ Nevertheless, the clinical interpretation of these variants should be managed carefully; in particular, at the current time, the p.S640T and p.R2339Q lack proof of disease segregation and need additional evidence to support their pathogenicity. A further word of caution is necessary concerning the association of *KMT2B* pathogenic variants with hearing loss as it has only rarely been reported and has been identified only in one of the families shown here, leaving open the possibility of a coincidental association with the *KMT2B* variant. Conversely, non-dystonic phenotypes such as mild intellectual disability and short stature are already consolidated clinical features of *KMT2B*-related disease, and were also observed in Family B. These observations should prompt future detailed family studies to characterize underrecognized non-dystonic features associated with *KMT2B* mutations.^11^

Seven of the patients reported here presented with adult-onset dystonia. All of them had an involvement of the upper part of the body; in particular, a cranio-cervical involvement (cervical dystonia, blepharospasm, oromandibular and laryngeal dystonia) was observed in 5 of 8 patients. The oromandibular and/or laryngeal regions are classically involved in childhood-onset KMT2B-related dystonia, sometimes leading to anarthria. No patient presented with predominant lower limb dystonia, which is instead classically observed in paediatric patients. In 3 of 8 patients, phasic upper limb dystonia and/or dystonic tremor developed during the disease course. One patient (B.II.3) exhibited overt oculomotor apraxia, which has been seldom reported and may represent another clue to suspect *KMT2B* variants in the context of segmental/generalized dystonia.^2^ None of the dystonic patients displayed a favourable response to oral anti-dystonic therapies (e.g. anticholinergics, benzodiazepines, tetrabenazine), whereas several of them received botulinum toxin injections with clinical benefit.

In conclusion, this report highlights the possible relevance of *KMT2B* missense variants as a genetic determinant of adult-onset dystonia and emphasizes the possibility of non-dystonic presentations of KMT2B-related disease. It is important to note that our findings represent an important first observation and need further confirmatory studies in independent pedigrees and cohorts. In this view, *KMT2B* gene should be considered in patients with adult-onset progressive dystonia involving the upper body part, in particular the larynx and oromandibular region and/or the upper limb. The identification of additional family members presenting with mild or even non-neurological phenotypes may represent an important clue to suggest this specific genetic aetiology.

## Supplementary Material

fcac276_Supplementary_Data

## Abbreviations

DNAm =: DNA methylation
MDS =: multidimensional scaling
SVM =: support vector machine
WES =: whole-exome sequencing
