# Supplementary material for: Adult-onset KMT2B-related dystonia
Source: Brain Commun. 2022 Oct 26;4(6):fcac276. doi: 10.1093/braincomms/fcac276 (PMC9724767; doi:10.1093/braincomms/fcac276)
Supplement: fcac276_Supplementary_Data [file fcac276_Supplementary_Data.zip › Supplementary_table_legends.docx]

**Supplementary Table 1:** Samples analyzed by means of DNAm array for the study cohort. For each sample, age, sex, and SVM score according to DYT28 classifier are reported. An SVM score > 0.5 is generally considered as minimum threshold for positive association with the considered trait.

**Supplementary Table 2:** List of the most informative differentially methylated CpG probes shared by the analysed subjects with late-onset dystonia and KMT2B variants.
